# Prevalence and physical characteristics of locomotive syndrome stages as classified by the new criteria 2020 in older Japanese people: results from the Nagahama study

**DOI:** 10.1186/s12877-021-02440-2

**Published:** 2021-09-09

**Authors:** Masashi Taniguchi, Tome Ikezoe, Tadao Tsuboyama, Yasuharu Tabara, Fumihiko Matsuda, Noriaki Ichihashi, on behalf of the Nagahama Study group

**Affiliations:** 1 Human Health Sciences, Graduate School of Medicine, Kyoto University, 53, Kawahara-cho, Shogoin, Sakyo-ku, Kyoto, 606-8507 Japan; 2 Department of Physical Therapy, School of Health Sciences, Bukkyo University, 7, Higashitogano-cho, Nishinokyo,Nakagyo-ku, Kyoto, 604-8418 Japan; 3 Center for Genomic Medicine, Graduate School of Medicine, Kyoto University, 53, Kawahara-cho, Shogoin, Sakyo-ku, Kyoto, 606-8507 Japan

**Keywords:** Locomotive syndrome, Prevalence, Physical performance, The 25-question geriatric locomotive function scale

## Abstract

**Background:**

The Japanese Orthopaedic Association (JOA) proposed the concept of locomotive syndrome (LS) in 2007 for detecting high-risk individuals with mobility limitation. In 2020, the JOA revised the clinical decision limits and introduced LS stage 3, which carried the highest-risk for LS compared to the conventional stages, 1 and 2. The purpose of this study was to characterize the prevalence, comorbidities, and physical characteristics in each LS stage, as per the LS criteria 2020.

**Methods:**

We analyzed 2077 participants (64.9% women; mean age, 68.3 ± 5.4 years) from the Nagahama Study aged ≥60 years. Participants were classified into 4 groups, non-LS and LS stages 1, 2, and 3, based on a 25-question Geriatric Locomotive Function Scale. The prevalence of comorbidities (sarcopenia, osteoporosis, diabetes mellitus, low back pain [LBP], and knee pain) were investigated. Physical characteristics were measured based on the physical performance tests including gait speed, five-times chair-stand, single-leg stand, and short physical performance battery; muscle strength tests including grip, knee extension, hip flexion, and abduction; and body-composition analysis including muscle quantity and quality. Differences in the prevalence of comorbidities between LS stages were tested using the chi-square test. The general linear model was performed for univariate and multivariate analyses with post-hoc test to compare the differences in physical characteristics among the LS stages.

**Results:**

The prevalence of LS increased with age, and the mean prevalence of LS stages 1, 2, and 3 were 24.4, 5.5, and 6.5%, respectively. The prevalence of comorbidities, including sarcopenia, osteoporosis, LBP, and knee pain, increased with worsening LS stage. Physical performance tests were significantly different between LS stages 2 and 3; and muscle strength differed significantly between LS stages 1 and 2. Additionally, in terms of body composition analysis, muscle quality but not muscle quantity showed significant differences among all the LS stages.

**Conclusions:**

Our findings suggest that muscle strengthening and dynamic training, including balance training in LS stage 1 and 2, respectively, were needed for preventing the LS progression. Individuals with LS stage 3 should perform dynamic training and muscle strengthening exercises while receiving treatment for comorbidities.

**Supplementary Information:**

The online version contains supplementary material available at 10.1186/s12877-021-02440-2.

## Background

Age-related decline in physical function is associated with disability in activities of daily living (ADL) and poor quality of life [1–3]. It is well-known that many of the older adults, who receive health care services, have problems of locomotive organs due to senility, falls/fractures, dementia, joint disorders, etc. [4]. Thus, detection of high-risk individuals is required to prevent and treat locomotive dysfunction. Based on this background, in 2007, the Japanese Orthopaedic Association (JOA) proposed the concept of “locomotive syndrome (LS)” to designate a state of mobility limitation with locomotive disorders. LS was judged by pain, ADL status, social functions, and mental health status, regardless of body weight or muscle mass loss. Thus, LS indicates a decline in physical function in a broader sense than that of sarcopenia and/or frailty. In fact, nearly all older people with sarcopenia and/or frailty also have coexisting LS [5]. Since assessing LS can help detect the disease and subsequent disability early, the concept of LS is expected to be increasingly adopted in countries with growing proportions of older adults. Several reports on LS have been recently published in international journals [6–8], and the LS assessment has also been used for older people outside Japan [9].

LS risk assessment is based on the clinical decision limit criteria decided by two physical tests and one self-administered questionnaire. The 25-question Geriatric Locomotive Function Scale (GLFS-25), which is a self-administered questionnaire, consists of questions on pain, ADL, social functions, and mental health status during the last month [10]; and has been used as a simple screening tool for LS risk assessment in many previous reports [11–13]. In the original version of LS, the total GLFS-25 scores ≥7 and ≥ 16 were classified into LS stage 1 and stage 2, respectively [14]. LS stage 1 was defined as a state when mobility functions start declining, and LS stage 2 was marked as the beginning of disease progression. It was reported that the estimated number of individuals, aged ≥40 years, with LS stage 2 was approximately 13.8 million in the Japanese population [15]. However, LS stage, which is likely to require medical attention, has not been defined previously. In 2020, JOA revised the clinical decision limits and introduced a new LS stage, stage 3. In the new classification of LS stages (the LS criteria 2020), LS stage 2 is defined by scores of 16 or more and less than 24 in the GLFS-25, and LS stage 3 by scores no less than 24 [16]. Since LS stage 3 is known to adversely affect social life, the Locomotive Challenge Council, established by the JOA works for developing LS countermeasures, has recommended a thorough medical examination by a specialist in these cases. Previous studies [17, 18], performed according to the LS criteria 2015, have reported that the prevalence of LS increased with age, and the prevalence was higher among women than among men. However, to the best of our knowledge, the prevalence of each stage as per the LS criteria 2020 has not been studied in community-dwelling older adults.

The risk factors for LS include decline in physical function, including physical performance, pain, muscle strength, and changes in body-composition [8, 11, 19–21]. A previous report [5] indicated that most individuals who had frailty and sarcopenia had coexistent LS. Among all the reported risk factors, low physical performance, as detected by usual walking speed, sit-to-stand ability, and one-leg standing time, remarkably increased LS risk [17, 20]. Thus, the physical performance in LS stage 3 could be lower than that in LS stage 2. However, to our best knowledge, there are no reports characterizing the differences in physical characteristics between each LS stage as per LS criteria 2020. Clarification of the decline in specific physical characteristics in each LS stage may provide valuable information for development of effective prevention strategies and therapies tailored to the LS stages.

The purpose of the present study was to characterize the prevalence of and differences in physical characteristics of individuals between LS stages categorized as per the new LS criteria 2020. We hypothesized that the prevalence of LS stage 3 increases with age, and women have a higher prevalence of having LS stage 3 than men. We also hypothesized that the physical characteristics in LS stage 3, especially physical performance, are lower than those in LS stage 2.

## Methods

### Participants

**Fig. 1:**
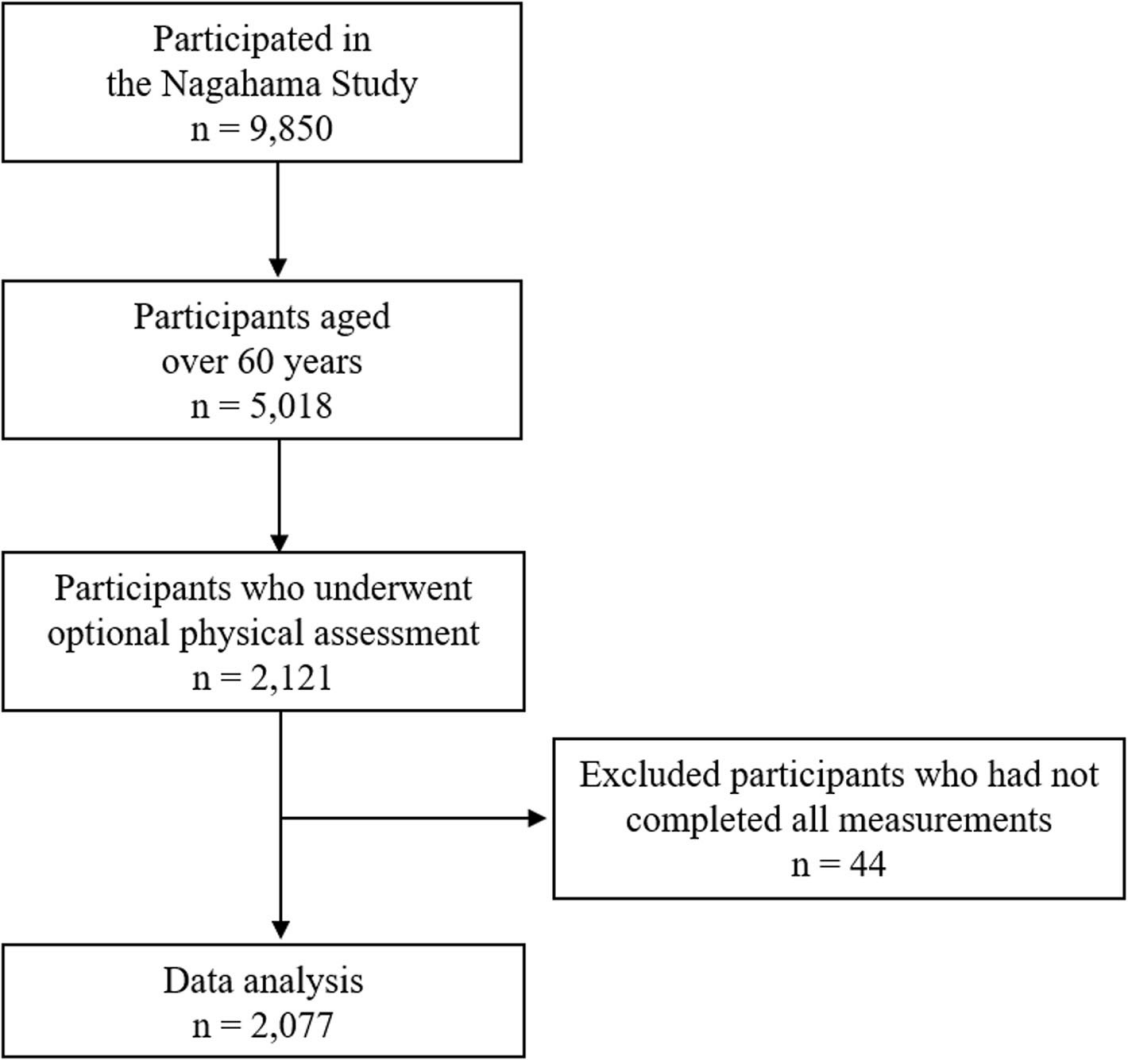
Flowchart for participants selection from the Nagahama Study

The cross-sectional study was conducted as a part of the second visit dataset of the Nagahama Prospective Cohort for Comprehensive Human Bioscience (herein referred to as the Nagahama study), which was a population-based cohort. Participant recruitment details are listed elsewhere [22]. Briefly, participants of this cohort aged 30–74 years at recruitment, lived independently without serious health problems, and were recruited via mass communications in the local community such as public relation magazines, newspapers, and personal solicitations. From the 9850 participants enrolled in this cohort, we provided additional explanations for the optional physical assessment in older adults aged ≥60 years. Among the 5018 participants aged 60 years or older, 2121 chose to undergo an optional physical assessment test. The inclusion criteria was the ability to walk at least 10 m. Participants who did not undergo all the measurements due to pain and fatigue and lacked data were excluded. Finally, we included 2077 participants for data analysis after excluding participants who had not completed all the measurements due to pain and fatigue (*n* = 44) (Fig. 1).

### Classification of locomotive syndrome

The GLFS-25 scores are self-reported measures, for which responses are recorded on a website. The GLFS-25 consists of 25 questions, including pain, disabilities of ADL, social functions, and mental health status during the last month. The total score of the GLFS-25 is a maximum of 100 points, and a high score indicates a lower locomotive function. Participants were classified into LS stages, as per the LS criteria 2020, on the basis of the total GLFS-25 scores as follows; total GLFS-25 scores ≥7 to < 16, ≥ 16 to < 24, and ≤ 24 were categorized as LS stage 1, stage 2, and stage 3, respectively [16].

### Physical performance measurement

Physical performance was measured by well-trained physical therapists using the following tests: usual gait speed, five-times chair-stand, single-leg standing, and short physical performance battery (SPPB). The usual gait speed was assessed by measuring the time taken to walk on a 10 m path, using a wireless phototube (Brower Timing Systems, Co., Ltd., UT, USA), along with simultaneous 4-m gait speed assessment. The five-times chair-stand test was evaluated using a standard chair with 40 cm height without an armrest, and participants were asked to stand up and sit down as fast as possible 5 times with their arms crossed. The single-leg standing time was measured twice in the participant’s selected leg, up to a maximum of 60 s and with eyes open. The better time of the two trials was used for analysis. Moreover, the static balance tests, including the quiet-standing with foot closed, semi-tandem, and tandem-standings, were carried out; and the SPPB scores were calculated by adding the results of 4-m gait speed and five-times chair-stand tests. The maximum possible score of the SPPB was 12 points, and the lower scores (≤ 9 points) represented worse physical function levels [23].

### Muscle strength measurement

The grip strength was measured in both hands using a standard digital grip dynamometer (Grip-D; Takei Scientific Instrument, Japan or YD; TTM, Japan). This measurement was performed in a sitting position with the participant’s arm positioned vertical to the ground. Both hands were measured twice, and the maximum value of all measurements was obtained for the analysis. In addition, muscle strengths of knee extension, hip flexion, and hip abduction in the right leg were measured during maximum voluntary isometric contraction. Participants were seated on a dynamometer (Musculater; OG Giken, Japan) with knee and hip joints in 90 degrees flexion for measuring the knee extension strength. In same position, hip flexion strength was measured using a hand-held dynamometer (Mobile MT-100; SAKAI Medical, Japan) by setting the sensor on the anterior surface of the distal thigh. Furthermore, hip abduction strength was measured in supine position with the knee fully extended using the hand-held dynamometer (Mobile MT-100; SAKAI Medical, Japan) while a force sensor set on 5-cm proximal from the lateral malleolus of the right leg. Participants were asked to perform these measurements twice, and the maximum force values (N) in each muscle strength were obtained and used for the analysis. Each force value was converted into torque (Nm) by multiplying with the thigh and shank length (m). Well-trained physical therapists performed these measurement tasks. The detailed description for muscle strength measurements has been indicated elsewhere [24].

### Body-composition analysis

Body-composition analysis was performed using a multifrequency bioelectrical impedance analysis (BIA) device (InBody 430, InBody Co., Ltd., Seoul, Korea). The BIA device with eight-point tactile electrode method obtains the resistance and reactance at 3 specific frequencies (5, 50, and 250 kHz) of the arms, trunk, and legs. The BIA technique is a valid tool for the assessment of body-composition, showing a good correlation with the dual-energy X-ray absorptiometry [25, 26]. The present study used the appendicular skeletal muscle mass, which was calculated automatically using a multifrequency BIA device, and was divided by the square of the individual’s height to obtain the value of skeletal muscle index (SMI; in kg/m^2^). Multi-frequency BIA can separately distinguish intracellular water (ICW) and extracellular water (ECW) from the total water. It is known that ICW, which reflects muscle cell mass is an indicator of muscle quantity, and ECW reflects non-contractile tissue including adipose tissue and interstitial fluid in the extracellular space. Higher ECW/ICW ratio means a relative increase of non-contractile tissue to muscle mass, which is an index of low muscle quality. Based on the protocols in a previous report [27], the ECW/ICW ratio was calculated using the impedance values at frequencies of 5 and 250 kHz.

### Clinical features and comorbidities

Height and body weight were measured to the nearest 0.1 cm and 0.1 kg and converted to body mass index (BMI, kg/m^2^). The presence of sarcopenia was diagnosed by assessing each individual’s SMI, grip strength, and/or the five-times chair-stand test using the Asian Working Group for Sarcopenia 2019 algorithm [28]. In addition, the presence of osteoporosis, diabetes mellitus, low back pain (LBP), and knee pain were confirmed by reviewing the self-reported questionnaire. LBP was defined based on continuous back pain for more than 3 months until the present date. Knee pain was also defined based on the occurrence of knee pain during the usual gait.

### Statistical analysis

Continuous variables were presented as means (standard deviations, SDs), and categorical variables were presented as numbers (percentages, %). All statistical analyses were performed using the SPSS software version 25.0 (SPSS Japan Inc., Tokyo, Japan). A *p*-value for the statistical significance was set at < 0.05.

The participants were divided into four age groups; 60–64 y, 65–69 y, 70–74 y, and over 75 y. Differences in the prevalence of each LS stage among four age groups were evaluated using the chi-square test and the chi-square test for trend, estimated using the entire study sample as well as sex-stratified sample. The general linear model was used for univariate analysis to test differences between four LS stages (the non-LS, LS stage 1, 2, and 3) in age and BMI, and post-hoc comparisons were conducted using a Bonferroni test. The chi-square test and the chi-square test for trend were also used to test the differences among the four LS stages in the prevalence of sarcopenia, osteoporosis, diabetes mellitus, LBP, and knee pain. The general linear model was performed for univariate and multivariate analyses to compare the differences among the four LS stages on the outcomes of physical performance, muscle strength, and body composition. Then, a post-hoc analysis with Bonferroni test was conducted to determine which LS stage differed significantly from the others. We also conducted multivariate analyses for the outcome measurements, adjusting for age, sex, and BMI.

## Results

### The prevalence of LS stages across the age groups

**Fig. 2:**
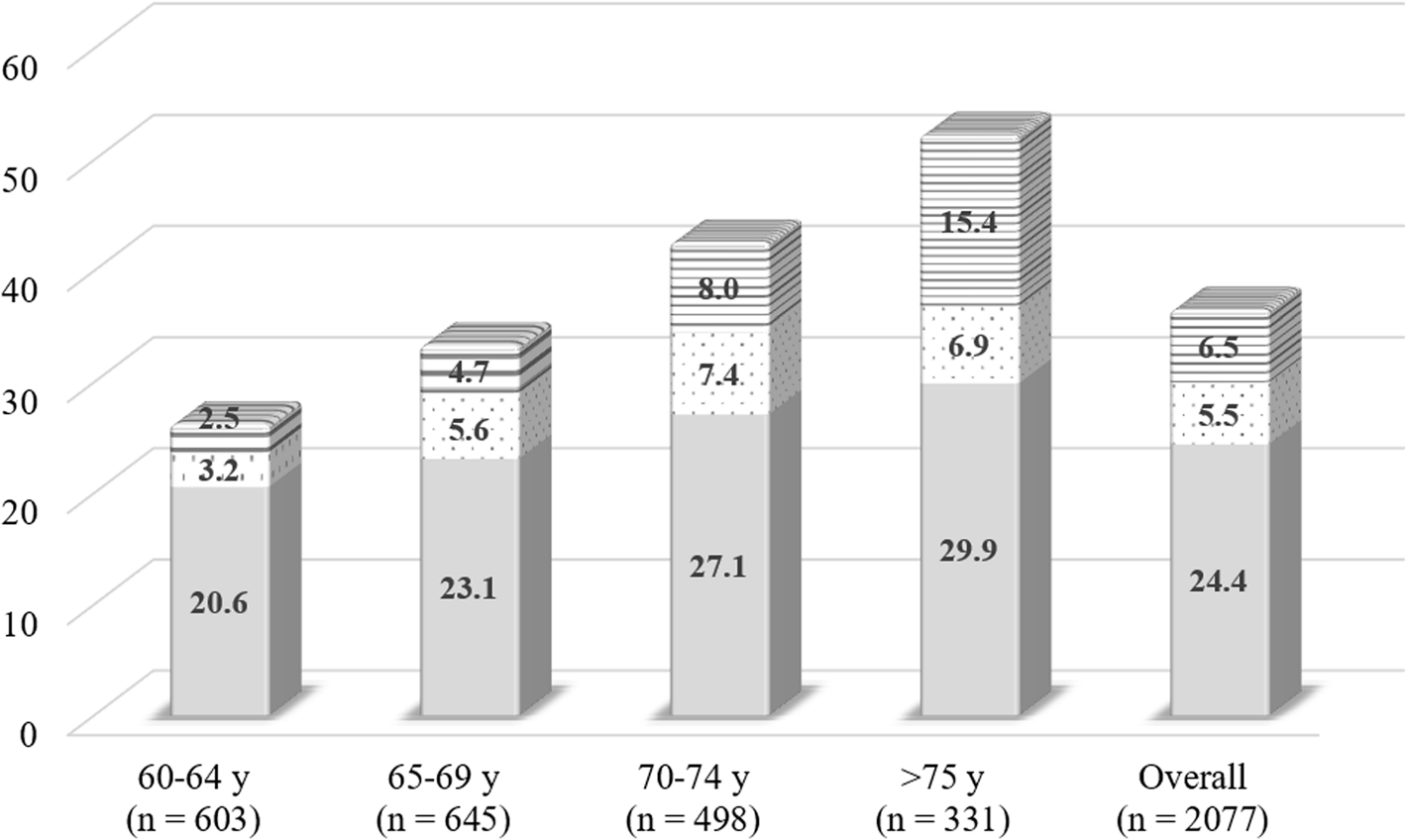
The prevalence of locomotive syndrome stages among the age groups in total sample (*n =* 2077). Gray bar, dot bar, and horizontal line bar indicate the presence of LS stage 1, stage 2 and stage 3, respectively. The values listed in the bar show the prevalence (%)

**Fig. 3:**
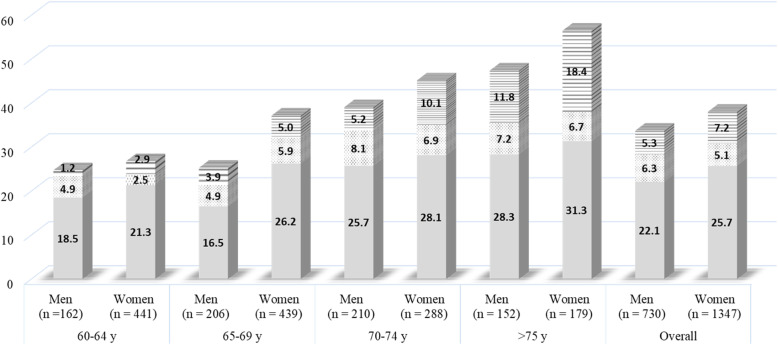
The prevalence of locomotive syndrome stages among the age groups in each sex-stratified sample. The prevalence of LS stage 1 (gray bar), stage 2 (dot bar), and stage 3 (horizontal line bar) among various age groups in men (*n =* 730) and women (*n =* 1347). The values listed in the bar show the prevalence (%)

The mean age of the study participants was 68.3 (5.4) years, and 1347 (64.9%) participants were women (Table 1). The prevalence of all LS stages significantly increased with age (p for trend, *p* < 0.001) (Fig. 2). Overall, the prevalence of LS stages 1, 2, and 3 was 24.4, 5.5, and 6.5%, respectively. Chi-square test showed a significant difference in the prevalence of each LS stage among age groups, and the prevalence of higher LS stages decreased in age groups 60–64 years and 65–69 years; while for the age-groups 70–74 years and > 75 years, the prevalence of LS stage 3 was higher than that of LS stage 2. In sex-stratified samples, the prevalence of all the LS stages increased significantly with age in both men and women (p for trend, *p* < 0.001) except for LS stage 2 in women (Fig. 3). Among men in the age group > 75 years, the proportion of LS stage 3 (11.8%) was higher than that of stage 2 (7.2%). Also, in the age groups 70–74 years and > 75 years in women, the proportions of participants in LS stage 3 (10.1 and 18.4%, respectively) were higher than those in LS stage 2 (6.9 and 6.7%, respectively).

**Table 1:** Demographics of the clinical features, comorbidities and physical characteristics in the study population

	Total	Men	Women
	*n* = 2077	*n* = 730	*n* = 1347
Age, y	68.3 (5.4)	69.5 (5.4)	67.7 (5.3)
BMI, kg/m^2^	22.4 (3.0)	23.0 (2.8)	22.0 (3.1)
GLFS-25 score, /total of 120	7.3 (9.2)	6.7 (8.4)	7.6 (9.6)
Prevalence of LS stage 1, n (%)	507 (24.4%)	161 (22.1%)	346 (25.7%)
stage 2, n (%)	115 (5.5%)	46 (6.3%)	69 (5.1%)
stage 3, n (%)	136 (6.5%)	39 (5.3%)	97 (7.2%)
Sarcopenia, n (%)	111 (5.3%)	49 (6.7%)	62 (4.6%)
Osteoporosis, n (%)	305 (14.7%)	10 (1.4%)	295 (21.9%)
Diabetes mellitus, n (%)	232 (11.2%)	119 (16.3%)	113 (8.4%)
LBP, n (%)	1133 (54.5%)	403 (55.2%)	730 (54.2%)
Knee pain, n (%)	802 (38.6%)	294 (40.3%)	508 (37.7%)
SMI, kg/m^2^	6.63 (0.94)	7.57 (0.68)	6.13 (0.60)
ECW/ICW ratio, a.u	4.74 (0.66)	4.28 (0.53)	4.99 (0.58)
Usual gait speed, m/s	1.30 (0.18)	1.32 (0.17)	1.27 (0.19)
Five-times chair-stand, s	8.68 (2.59)	8.47 (2.37)	9.07 (2.92)
Single-leg standing, s	44.29 (20.02)	45.94 (19.19)	41.24 (21.13)
SPPB, /total of 12	11.72 (0.71)	11.78 (0.60)	11.61 (0.86)
Grip power, kg	28.35 (8.75)	37.92 (6.82)	23.16 (4.04)
Knee extension strength, Nm	116.64 (50.19)	159.35 (53.04)	93.50 (28.90)
Hip flexion strength, Nm	41.98 (15.65)	53.28 (17.62)	35.86 (10.14)
Hip abduction strength, Nm	24.29 (7.89)	30.48 (7.82)	20.94 (5.55)

The continuous variables were shown as mean (SD), and categorical variables as frequencies (%)

*BMI* body mass index, *GLFS* geriatric locomotive function scale, *LS* locomotive syndrome, *LBP* low back pain, *SMI* skeletal muscle mass index, *ECW/ICW* extracellular-to-intracellular water, *SPPB* Short Physical Performance Battery

### Differences in the clinical features and comorbidities among the four study groups

The prevalence of sarcopenia, osteoporosis, LBP, and knee pain differed significantly among the LS stages (chi-square test; *p* value < 0.05) (Table 2). The proportion of sarcopenia increased in higher LS stages, and the proportions of osteoporosis and knee pain were higher in LS stage 3 than in other LS stages. On the other hand, the proportion of LBP in LS stages, 1, 2, and 3 was remarkably higher (76.1, 87.0, and 88.2%, respectively) than that in the non-LS group (40.0%).

**Table 2:** Clinical features and comorbidities among locomotive syndrome stages

	Non-LS	LS stage 1	LS stage 2	LS stage 3	*p*-value	P for trend/Post-hoc test
	*n* = 1319	*n* = 507	*n* = 115	*n* = 136		
GLFS-25 score, /total of 100	2.5 (1.9)	9.8 (2.5)	19.0 (2.3)	34.4 (11.1)	n/a	n/a
Sex: women, n (%)	835 (62.0%)	346 (68.2%)	69 (60.0%)	97 (71.3%)	< 0.001	0.086
Age, y	67.5 (5.2)	69.1 (5.4)	69.8 (5.3)	71.9 (5.2)	< 0.001	1, 2, 3, 5, 6
BMI, kg/m^2^	22.1 (2.8)	22.5 (3.0)	23.4 (3.7)	23.8 (4.0)	< 0.001	2, 3, 5
Sarcopenia, n (%)	42 (3.2%)	40 (7.9%)	10 (8.7%)	19 (14.0%)	< 0.001	< 0.001
Osteoporosis, n (%)	144 (10.9%)	100 (19.7%)	21 (18.3%)	40 (29.4%)	< 0.001	< 0.001
Diabetes mellitus, n (%)	131 (9.9%)	63 (12.4%)	18 (15.7%)	20 (14.7%)	0.076	0.012
LBP, n (%)	527 (40.0%)	386 (76.1%)	100 (87.0%)	120 (88.2%)	< 0.001	< 0.001
Knee pain, n (%)	415 (31.5%)	252 (49.7%)	55 (47.8%)	80 (58.8%)	< 0.001	< 0.001

The continuous variables were shown as mean (SD), and categorical variables as frequencies (%). Chi-square test was conducted for sex and comorbidities (sarcopenia, osteoporosis, diabetes mellitus, LBP, and knee pain) and also confirmed p for trend. The general linear model and post-hoc analysis with Bonferroni test was performed to compare the differences among the four LS stages on age and BMI. The number indicates a significant difference by the post hoc test: 1, Non-LS versus LS stage 1; 2, Non-LS versus LS stage 2; 3, Non-LS versus LS stage 3; 4, LS stage1 versus stage 2; 5, LS stage 1 versus stage 3; 6, LS stage 2 versus stage 3

*LS* locomotive syndrome, *GLFS* geriatric locomotive function scale, *BMI* body mass index, *LBP* low back pain

### Differences in the physical performance, muscle strength and body-composition among the four study groups

Significant differences among the four LS groups were found in all the physical performance and muscle strength tests, and the ECW/ICW ratio, except for the SMI (Table 3). The results of post-hoc test in all the physical performance tests and ECW/ICW ratio showed significant differences among all LS groups, except between LS stages 1 and 2. In the lower-limb muscle strength tests, there were significant differences between the non-LS group and the 3 LS stage groups. After adjusting for age, sex, and BMI, significant differences were found in all the physical performance tests between the LS groups, except between LS stages 1 and 2. In muscle strength tests, knee extension and hip abduction strength test showed significant differences between all the LS groups, except between LS stages 2 and 3. Moreover, the ECW/ICW ratio showed significant differences between all the four study groups.

**Table 3:** Differences in the physical performance, muscle strength and body-composition among locomotive syndrome stages

	Non-LS	LS stage 1	LS stage 2	LS stage 3	*F*-value	*p*-value	Post-hoc test	
	*n =* 1319	*n =* 507	*n =* 115	*n =* 136			Univariate	Multivariate
Usual gait speed, m/s	1.33 (0.17)	1.26 (0.17)	1.24 (0.18)	1.13 (0.21)	71.06	< 0.001	1, 2, 3, 5, 6	1, 2, 3, 5, 6
Five-times chair-stand, s	8.25 (2.23)	9.07 (2.60)	9.66 (2.84)	10.6 (3.89)	49.47	< 0.001	1, 2, 3, 5, 6	1, 2, 3, 5, 6
Single-leg standing, s	47.37 (18.26)	41.47 (20.98)	37.79 (22.06)	30.43 (22.19)	41.87	< 0.001	1, 2, 3, 5, 6	1, 2, 3, 5, 6
SPPB, /total of 12	11.82 (0.52)	11.66 (0.76)	11.49 (0.97)	11.17 (1.30)	44.18	< 0.001	1, 2, 3, 5, 6	1, 2, 3, 5, 6
Grip power, kg	29.23 (8.82)	27.07 (8.38)	27.50 (9.00)	25.29 (7.83)	14.31	< 0.001	1, 3	1, 2, 3
Knee extension strength, Nm	121.28 (50.95)	111.52 (47.52)	108.52 (47.90)	97.63 (47.46)	13.25	< 0.001	1, 2, 3, 5	1, 2, 3, 4, 5
Hip flexion strength, Nm	43.96 (16.15)	39.49 (14.23)	37.73 (13.23)	35.73 (14.02)	22.01	< 0.001	1, 2, 3	1, 2, 3
Hip abduction strength, Nm	25.36 (7.78)	22.99 (7.55)	22.61 (8.03)	20.20 (7.79)	27.68	< 0.001	1, 2, 3, 5	1, 2, 3, 4, 5
SMI, kg/m^2^	6.65 (0.93)	6.57 (0.90)	6.77 (1.04)	6.59 (1.00)	1.94	0.121	–	–
ECW/ICW ratio, a.u	4.66 (0.64)	4.81 (0.62)	4.84 (0.66)	5.13 (0.78)	25.84	< 0.001	1, 2, 3, 5, 6	1, 2, 3, 4, 5, 6

The general linear model was used for univariate and multivariate analyses, and post-hoc test was performed to assess the differences among the groups. The values of *F*-value and *p*-value represent the results of univariate analysis. The number indicates a significant difference by the post hoc test: 1, Non-LS versus LS stage 1; 2, Non-LS versus LS stage 2; 3, Non-LS versus LS stage 3; 4, LS stage1 versus stage 2; 5, LS stage 1 versus stage 3; 6, LS stage 2 versus stage 3

*LS* locomotive syndrome, *SPPB* short physical performance battery, *SMI* skeletal muscle mass index, *ECW/ICW* extracellular-to-intracellular water

## Discussion

To the best of our knowledge, the present study is the first to clarify the prevalence of LS stages as defined by new criteria 2020 and the differences in physical characteristics between these new LS stages. LS criteria 2020 classified the individuals in the conventional LS stage 2 into LS stage 2 and 3. Partially in agreement with our hypothesis, the prevalence of LS stage 2 and 3 was found to increase with age; and the proportion of LS stage 3 was higher in women than in men for all age strata. Interestingly, the proportions of LS stage 3 in the age groups > 70 years in women and > 75 years in men were higher than that of LS stage 2. The prevalence of comorbidities including sarcopenia, osteoporosis, LBP, and knee pain was high in LS stage 3. Furthermore, the significant group differences between LS stage 2 and 3 were observed in the physical performance tests, while between LS stage 1 and 2, significant differences were observed in the knee extension and hip abduction strength tests. These results suggested that differences in physical characteristics between LS stage 1 and 2 were due to muscle weakness of knee extension and hip abduction, and between LS stage 2 and 3 were due to declining physical performance. LS stage 3 patients had a higher prevalence of comorbidities and experienced a decline in physical performance. These findings provide valuable insights that may help develop appropriate training modality necessary for each stage.

A previous study reported by Seichi et al. [18] showed that the overall mean prevalence of LS stage 2, defined by conventional criteria (total GFLS-25 score > 16), was 11.9% (*n* = 9027; age, 40–70 years). In the present study, the mean prevalence of conventional LS stage 2 among participants aged > 60 years was approximately 12.0% (5.5% for new LS stage 2 and 6.5% for new LS stage 3, classified by LS criteria 2020). Our results highlighted a new finding of higher prevalence of LS stage 3 compared to that of stage 2. This pattern was particularly evident in women aged > 70 y and in men aged > 75 y, implying that the proportion of high-risk individuals with a decline in locomotive function was high in older age groups. In accordance with previous studies [17, 18], we observed an age-related increase in LS prevalence among both men and women. The age-related increase was particularly remarkable among women, with the prevalence of LS stage 3 increasing from 2.9% in the age group 60–64 years to 18.4% in the age group > 75 years. Although it is known that female sex is one of the risk factors for LS [6], it could be confounded by the presence of osteoporosis [29]. In fact, this study indicated that the overall prevalence of osteoporosis in women was 21.9%, but was 1.4% in men; the proportion being the highest in LS stage 3. Although Yoshimura et al. [5] have reported that co-existence of sarcopenia with conventional LS stage 2 was approximately 7.0%, the present study showed that co-existence of sarcopenia with LS stages 2 and 3 was 8.7 and 14.0%, respectively. As both sarcopenia and LS are accompanied by decreased physical function, their coexistence may be more strongly associated with a decline in physical function. Moreover, the proportion of LBP in all the three LS stages was remarkably high (76.1 to 88.2%) as compared to that in the non-LS group. LBP has already been recognized as a risk factor for LS [29]. This attribution has not changed following the revision of the LS criteria. In contrast, the current study confirmed that the proportions of osteoporosis and knee pain in LS stage 3 (osteoporosis, 29.4%; knee pain, 58.8%) were higher than those in LS stage 2 (osteoporosis, 18.3%; knee pain, 47.8%), thus the presence of osteoporosis and knee pain could be the features of high-risk individuals for LS stage 3.

Significant group differences in the physical performance tests were observed between LS stages 2 and 3, after adjusting for age, sex, and BMI. These findings supported our hypothesis that individuals with LS stage 3 have poorer physical performance than those with LS stage 2. The revision of the LS criteria successfully classified individuals with lower physical performance into LS stage 3. In contrast, there was no significant group difference in the physical performance tests between LS stages 1 and 2. Since the individuals in the conventional LS stage 2 have been reclassified into LS stage 2 and 3 as per the LS criteria 2020, the new LS stage 2 could have relatively better physical performance than the conventional LS stage 2. After adjusting for age, sex, and BMI, knee extension and hip abduction strength test showed significant differences between LS stage 1 and stage 2, but not between LS stage 2 and 3. The present study also found that physical performance correlated with the lower-limb muscle strength; however, the relationship was very weak (Supplementary Table). This weak correlation, in turn, could affect the distinction of physical characteristics between LS stages. A previous study [30] has indicated that the annual decline rate in knee extension strength was greater than in usual walking speed in the older individuals. Therefore, assessment of muscle strength may be useful for early detection of LS stage 2, which is the pre-stage of evident decline in physical performance. Interestingly, worsening of LS stage increased the prevalence of sarcopenia and induced loss of physical function in terms of muscle strength affecting the overall performance. According to the revised sarcopenia diagnosis guidelines [31], severe sarcopenia is diagnosed when physical performance decline is observed in addition to muscle weakness and loss of muscle mass. Furthermore, significant group differences in ECW/ICW ratio were demonstrated among all the groups. Tanaka et al. [8] have suggested that this indicator is strongly associated with LS risk, consistent with our results. Higher ECW/ICW ratios reflect a relative increase in non-contractile tissue to skeletal muscle, thus acting as a biomarker for the loss of muscle quality [32]. Recently, higher ECW/ICW ratios, but not SMI, were found to be associated with severe functional disability in patients with knee OA [33]. Therefore, muscle quality indicator measured by multiple-frequency BIA may also be useful for assessing the locomotive function.

The Locomotive Challenge Council recommends locomotion training, known as “locotra”, which consists of single-leg standing, squatting, and muscle strengthening exercises of the trunk and quadriceps [16]. These four programs conduct no-equipment calisthenics exercise training sessions, with 3 sets per day and twice a week, and correspond to more than 3 METs for approximately 30 min. As clinical implications, the results of our study demonstrate the necessity for muscle strengthening in LS stage 1 and dynamic training including balance in LS stage 2 to prevent the progression of locomotive dysfunction. Therefore, these findings provide additional information on training contents which should be highlighted in each LS stage. The present study indicated that many individuals with LS stage 3 had a higher prevalence of comorbidities and decline in physical performance. Given this fact, individuals with LS stage 3 should perform dynamic training and muscle strengthening in parallel to receiving treatment for comorbidities.

The present study had several limitations. First, our focus on Nagahama Study participants who opted for optional physical examinations is a possible source of selection bias. The medical education and treatment status of the participants in this study was unknown. If they had thoroughly undergone the optional physical examination, they could have had a good physical function. Secondly, since the prevalence of each LS stage was based on the total GLFS-25 score, the results cannot be extended to the prevalence based on stand-up test and two-step test. The stand-up test is used to assess leg strength by the participant standing up on single or both legs from seats at four specified heights; 40, 30, 20, and 10 cm. The two-step test is also used to assess leg strength, balance, and flexibility by measuring the maximum length of the double stride the participant can step forward [16]. The LS categorization based on GLFS-25 score was found to be sensitive than that based on the two physical tests [34], thus we assumed that similar results will be observed irrespective of the evaluation tests performed. The third limitation of this study was that due to the cross-sectional design of the study, the threshold for progression of LS stage in relation to each outcome measurement was not clear. Future longitudinal studies are needed to clarify the risk factors and their thresholds for predicting LS progression.

## Conclusions

Among the LS stages as per LS criteria 2020, the prevalence of LS stage 2 and 3 increased with age, and the proportion of LS stage 3 among women was higher than that among men. Among the physical characteristics, the physical performance tests varied between LS stages 2 and 3, and the knee extension and hip abduction strength varied between LS stages 1 and 2. Our findings suggested that muscle strengthening in LS stage 1 and dynamic training including balance training in LS stage 2 were needed for preventing the progression of locomotive dysfunction. Individuals with LS stage 3 should receive dynamic training and muscle strengthening training while undergoing treatment for comorbidities.

## Supplementary Information

**Additional file 1: Supplementary Table.** Results of correlation analysis between physical performance tests and muscle strength tests using Pearson’s correlation analysis.

## Data Availability

This data set is still being used for analysis. Please contact the corresponding author regarding access to the full dataset.

## Abbreviations

ADL: Activities of daily living
JOA: Japanese Orthopaedic Association
LS: Locomotive syndrome
GLFS-25: The 25-question geriatric locomotive function scale
SPPB: Short physical performance battery
BIA: Bioelectrical impedance analysis
SMI: Skeletal muscle index
ICW: Intracellular water
ECW: Extracellular water
BMI: Body mass index
LBP: Low back pain
SD: Standard deviations
