# Supplementary material for: Prevalence and physical characteristics of locomotive syndrome stages as classified by the new criteria 2020 in older Japanese people: results from the Nagahama study
Source: BMC Geriatr. 2021 Sep 9;21:489. doi: 10.1186/s12877-021-02440-2 (PMC8428127; doi:10.1186/s12877-021-02440-2)
Supplement: Supplementary file 1 — Additional file 1: Supplementary Table. Results of correlation analysis between physical performance tests and muscle strength tests using Pearson’s correlation analysis. [file 12877_2021_2440_MOESM1_ESM.docx]

**Supplementary Table.** Results of correlation analysis between physical performance tests and muscle strength tests using Pearson’s correlation analysis.

|  | Usual gait speed | Five-times chair-stand | Single-leg standing | SPPB | Grip power | Knee extension strength | Hip flexion strength | Hip abduction strength |
| --- | --- | --- | --- | --- | --- | --- | --- | --- |
| Usual gait speed | - |  |  |  |  |  |  |  |
| Five-times chair-stand | -0.346** | - |  |  |  |  |  |  |
| Single-leg standing | 0.191** | -.0274** | - |  |  |  |  |  |
| SPPB | 0.414** | -0.715** | 0.243** | - |  |  |  |  |
| Grip power | 0.02 | -0.083** | 0.046* | 0.042 | - |  |  |  |
| Knee extension strength | 0.080** | -0.144** | 0.067** | 0.105** | 0.721** | - |  |  |
| Hip flexion strength | 0.064** | -0.175** | 0.105** | 0.103** | 0.610** | 0.667** | - |  |
| Hip abduction strength | 0.110** | -0.153** | 0.077** | 0.120** | 0.655** | 0.641** | 0.617** | - |

Statistical significance: * p ˂ 0.05, ** p ˂ 0.01

SPPB, short physical performance battery.
